# Metallothionein 2A inhibits NF-κB pathway activation and predicts clinical outcome segregated with TNM stage in gastric cancer patients following radical resection

**DOI:** 10.1186/1479-5876-11-173

**Published:** 2013-07-19

**Authors:** Yuanming Pan, Jiaqiang Huang, Rui Xing, Xin Yin, Jiantao Cui, Wenmei Li, Jun Yu, Youyong Lu

**Affiliations:** 1 Laboratory of Molecular Oncology, Key laboratory of Carcinogenesis and Translational Research, Ministry of Education, Peking University Cancer Hospital & Institute, No.52 Fucheng Road, Beijing, Haidian District 100142, People's Republic of China; 2 College of Life Sciences & Bioengineering, School of Science, Beijing Jiaotong University, No.3 ShangyuanCun, Beijing, Haidian District 100044, People's Republic of China; 3 Institute of Digestive Disease and Department of Medicine and Therapeutics, Li Ka Shing Institute of Health Sciences, The Chinese University of Hong Kong, Hong Kong, SAR, China

**Keywords:** Metallothionein 2A, NF-κB signaling, Gastric cancer, Prognosis, TNM stage

## Abstract

**Background:**

Metallothionein 2A (MT2A) as a stress protein, plays a protective role in gastric mucosal barrier. Its role in the development of gastric cancer (GC) is unclear. The mechanism of MT2A will be investigated in gastric tumorigenesis.

**Methods:**

MT2A expression was detected in 973 gastric specimens. The biological function was determined through ectopic expressing MT2A *in vitro* and *in vivo*. The possible downstream effectors of MT2A were investigated in NF-κB signaling. The protein levels of MT2A, IκB-α and p-IκB-α (ser32/36) expression were analyzed in a subset of 258 patients by IHC staining. The prognostic effects of MT2A, status of IκB-α and TNM stage were evaluated using the Kaplan-Meier method and compared using the log-rank test.

**Results:**

Decreased MT2A expression was detected in cell lines and primary tumors of GC. In clinical data, loss of MT2A (MT2A + in Normal (n =171, 76.0%); Intestinal metaplasia (n = 118, 50.8%); GC (n = 684. 22.4%, *P* < 0.001)) was associated with poor prognosis (*P* < 0.001), advanced TNM stage (*P* = 0.05), and down-regulation of IκB-α expression (*P* < 0.001). Furthermore, MT2A was the independent prognostic signature segregated from the status of IκB-α and pathological features. In addition, MT2A inhibited cell growth through apoptosis and G2/M arrest, which negatively regulated NF-κB pathway through up-regulation of IκB-α and down-regulation of p-IκB-α and cyclin D1 expression.

**Conclusions:**

MT2A might play a tumor suppressive activity through inhibiting NF-κB signaling and may be a prognostic biomarker and potential target for individual therapy of GC patients.

## Background

### What’s new

Abundance of Metallothionein 2A (MT2A) exhibits in the gastric mucosal barrier, but its role in the progression of gastric cancer (GC) is still unclear. In this analysis of 171 normal gastric tissues, 118 intestinal metaplasia, and 684 primary gastric cancers, decreased MT2A was significantly correlated with poor prognosis. It inhibited the proliferation of GC cells through NF-κB signaling inactivation. These results indicate that MT2A may be an important prognostic marker and therapeutic target for GC.

Gastric cancer (GC) is the most commonly diagnosed malignancy and remains a significant burden of cancer in Asia, especially in China [1,2]. Most GC patients undergoing surgery are already at an advanced stage and the 5-year survival is varied. Several histological factors have been reported to be prognostic factors of GC, including tumor size, WHO classification, tumor-node-metastasis (TNM) stage system, and differentiation grade [3,4]. However, prognosis of GC patients at the same stage is still inconsistent [5]. Therefore, identification of specific diagnostic markers and therapeutic target would allow reliable prediction, effective extension of postoperative survival and life quality of patients [6].

Cellular stress has been shown to play a role in the molecular regulation of carcinogenesis [7,8]. Metallothioneins (MTs) as the stress proteins with low molecular weight and rich-cysteine have the ability of a high affinity for metal ions and ROS scavengers. MT2A as the main isoform of MTs plays an important role in gastric mucosal barrier in patients with gastritis and rodent models [9,10]. Pre-administration of exogenous MT2A or pre-induction of endogenous MT2A can protect stomach and liver against stress-induced damage and inhibit the formation of stress-induced lipid peroxide, implying a protective effect of MT2A on stress-induced pathogenesis and a potential therapeutic target applied for early prevention [11]– [13]. Recently, MT2A plays an important role in tumorigenesis and progression of multiple carcinomas including GC [14]. The mice loss of MT2A gene predisposed to diethylnitrosamine-induced hepatocarcinogenesis by activating NF-κB target genes, which demonstrates that MT2A protects mice from hepatocarcinogen-induced liver damage and carcinogenesis, underscoring its potential therapeutic application against hepatocellular cancer [15]. Some studies focused on the role of MT2A in the protection against *H.pylori*-induced gastric injury using MT-null mice. Himeno, S. discovered that activation of NF-κB and expression of NF-κB-mediated chemokines in gastric cells were markedly higher in MT2A-null mice than in wide-type mice [9]. These data imply that MT2A realizes negative control of the transcription factor NF-κB activity, but its role in gastric carcinogenesis is still ambiguous [16]– [18].

Aberrant activation of NF-κB is associated with cell inflammation, malignancy, and tumor progression [7,19]. The functional activity of NF-κB is inhibited through binding to its inhibitor, IκB-α. Activation of NF-κB is resulted from proteasome-mediated degradation of IκB-α by phosphorylation of the inhibitor (p-IκB-α), which suggests that NF-κB pathway is a potential target for individual therapy [20]– [23].

Some evidence indicated that increased MT2A expression is important for cancer progression, and MT2A is initially proposed as a proto-oncogene in breast, esophageal, prostate, and ovarian cancers, associated with malignancy and poor prognosis [24]– [27]. In contrast, it is down-regulated in gastrointestinal tumors and hepatocellular carcinomas, where MT2A is either inversely correlated or unrelated to mortality [28,29]. However, the variation of MT2A and its clinical evaluation remains contradictory in GC [28,30,31]. These results suggest that dysregulation of MT2A is involved in tumor pathogenesis, although the exact role is still unclear in GC.

Hence, we focused to reveal the co-expression of MT2A and IκB-α gene correlated with clinical pathological features and outcomes in a large scale of gastric tumors with long-term follow-up data. Furthermore, we systematically analyzed the role of MT2A as a stress protein and negative regulator in NF-κB activation to characterize its biological role and molecular mechanism *in vitro* and *in vivo*.

## Methods

### Patient characteristics

Gastric tumor samples from 684 GC patients treated at Beijing Cancer Hospital from 1997 to 2007 were assessed. The patients included 513 men (75.0%) and 171 women (25.0%) with an average age at diagnosis of 54 years (range, 19 to 81 years). The follow-up period ranged from 1 to 127.2 months (median, 34.2 months). All patients underwent radical resection with curative intent. None of the patients had received neoadjuvant chemotherapy or radiation therapy prior to surgery. The inclusion criteria of all the patients is: (a) a distinctive GC diagnosis based on the sixth edition of the tumor-node-metastasis (TNM) classification of the International Union against GC, (b) a radical resection, (c) suitable formalin fixed, paraffin embedded tissues. The total of 684 GCs had the clinicopathological data (termed as ‘cohort’). Moreover, a set of 258 eligible samples were collected from the cohort with 10-year follow-up data (termed as ‘subset’). The clinical outcome of the patients was recorded from the date of surgery to the date of death. In addition, 118 intestinal metaplasia (IM) and 171 normal gastric tissues were collected from these GC patients. Histological evaluation was performed by three senior pathologists. The demographic breakdown of the cohort and subset is listed in Supplementary Table 1. Besides, 36 paired gastric tumors with adjacent normal tissues was ground to a powder under liquid nitrogen for RT-PCR and Real-Time PCR of MT isoforms (Additional file 1).

**Table 1:** Logistic regression analysis of clinicopathological features and prognosis in GC

**Variates**	**No. of cases (n = 258)**	**Survival time (month)**		***P*-value**
Sex				
Male	186	66.4 ± 4.6		
Female	72	54.1 ± 4.8		0.604
Age at diagnosis				
<60	173	65.0 ± 4.9		
≥60	85	59.2 ± 5.6		0.867
TNM stage				
I	34	105.5 ± 7.7	I vs. IV	< 0.001
II	57	74.0 ± 5.9	II vs. IV	< 0.001
III	115	49.1 ± 3.4	III vs. IV	0.010
IV	52	35.8 ± 4.1		
Tumor depth				
pT1	13	87.2 ± 9.9	pT1 vs. pT4	0.005
pT2	50	89.1 ± 9.1	pT2 vs. pT4	< 0.001
pT3	157	49.1 ± 2.9	pT3 vs. pT4	0.159
pT4	38	40.1 ± 5.3		
Lymph node status				
pN0	78	89.8 ± 6.2	pN0 vs. pN3	0.001
pN1	115	50.5 ± 3.4	pN1 vs. pN3	0.212
pN2	49	46.6 ± 6.0	pN2 vs. pN3	0.326
pN3	16	47.5 ± 12.6		
Distant metastasis				
pM0	232	67.5 ± 4.3		
pM1	26	27.6 ± 5.8		< 0.001
Degree of differentiation				
D1	54	76.0 ± 6.0		
D0	204	59.8 ± 4.4		0.011
MT2A expression				< 0.001
low	202	53.9 ± 3.4		
high	56	86.9 ± 9.2		
IκB-α expression				
low	164	61.6 ± 4.9		
high	94	64.9 ± 5.1		0.248
p-IκB-α expression				
low	126	74.1 ± 5.3		
high	132	47.7 ± 3.4		0.005

M0: no distant metastasis; M1: distant metastasis;

D1: well/moderately-differentiated GCs; D0: poorly-differentiated GCs.

### Immunohistochemical analysis

IHC staining in tissue array was performed with MT2A antibody (1:100 dilution; 18-0133, Invitrogen, CA, US), p-IκB-α antibody (1:200 dilution; ab47752, Abcam, Cambridge, UK), IκB-α antibody (1:300 dilution; sc-371, Santa Cruz Biotechnology, CA). For each biomarker, images were scored visually by three pathologists who were blinded to clinical outcome. Discrepancies were resolved by consensus. Scores were assigned as a percentage of positive staining within each cylinder. The mean percentage value of the two cores was calculated to represent one tumor (Additional file 1).

### Cell lines

Gastric cancer cell lines BGC823, MGC803, SGC7901 and PAMC82 were established in China and purchased from the tissue bank of Shanghai (Shanghai, China). Especially, BGC823 cells exhibited high tumorigenecity. MKN45, AGS, N87, RF-1, RF-48, SNU-1, SNU-5 and SNU-16 cell lines were purchased from ATCC (American Type Culture Collection, Manassas, US). GES-1, an immortalized human gastric epithelial cell line, was generated by SV40 viral transfection at Beijing Cancer Hospital and cultured in DMEM medium supplemented with 10% fetal bovine serum (Gibco, Life technologies, Grand Island, NY, USA) at 37°C in a humidified atmosphere containing 5% CO_2_[32].

### MTT and soft agar assay

The cells were seeded into 96-well culture plates, and MTT was added to the cells at 1-5 days. MTT (5 μg/ml) was removed after 4 h incubation, and then dimethyl sulfoxide (DMSO) was added to solubilize the formazan product. The absorbency at 490 nm/570 nm was assayed by a microplate reader (Bio-rad680 ELISA). Cells were then incubated for 4 weeks, stained with vital tetrazolium dye INT (piodonitrotetrazolium, Invitrogen) to document the presence or absence of viable cell colonies. The soft agar was fixed with 100 μl methanol-acetic acid (3:1 vol/vol) and colonies were counted.

### Tumorigenicity assay

BGC823 cells (5 × 10^5^ cells suspended in 0.1 ml PBS) transfected with MT2A over-expressed vector or empty vector were injected subcutaneously into the dorsal flank of five 4-week-old female Balb/C nude mice (MT2A-expression clones on the right and vector control clones on the left. Tumor diameter was measured and documented every 5 days. Tumor volume was calculated according to the formula ab^2^/2 (a > b). At the end of 25 days, all mice were sacrificed and the tumor volume was measured. Three independent experiments were performed and gave the similar results. The animals were maintained in facilities approved by the association for assessment and accreditation of Laboratory Animal Care in accordance with the current regulations and international standards.

### Statistical analysis

*χ*^2^ test statistics and Student’s *t*-test were used to compare pretreatment characteristics of patient cases. The cancer-related survival was analyzed using the Kaplan–Meier method and compared using log-rank tests. The Spearman rank test and Fisher’s exact test were applied to demonstrate clinicopathological correlations. A Cox proportional hazard regression model was used with associated 95% confidence intervals (CIs) and *P* values. All statistical tests were two-sided, and *P* values of less than 0.05 were considered statistically significant. The statistical analysis was performed using the statistical package SPSS (Version 16.0; SPSS Inc, Chicago, IL).

### Study approval

All animal studies were approved by the Ethics Committee of Peking University Cancer Hospital. The use of human tissues and clinical data was according to the guidelines of the hospital and approved by the Local Ethical Committee.

## Results

### Decreased MT2A expression is a molecular event in cell lines and primary tumors of GC

**Figure 1:**
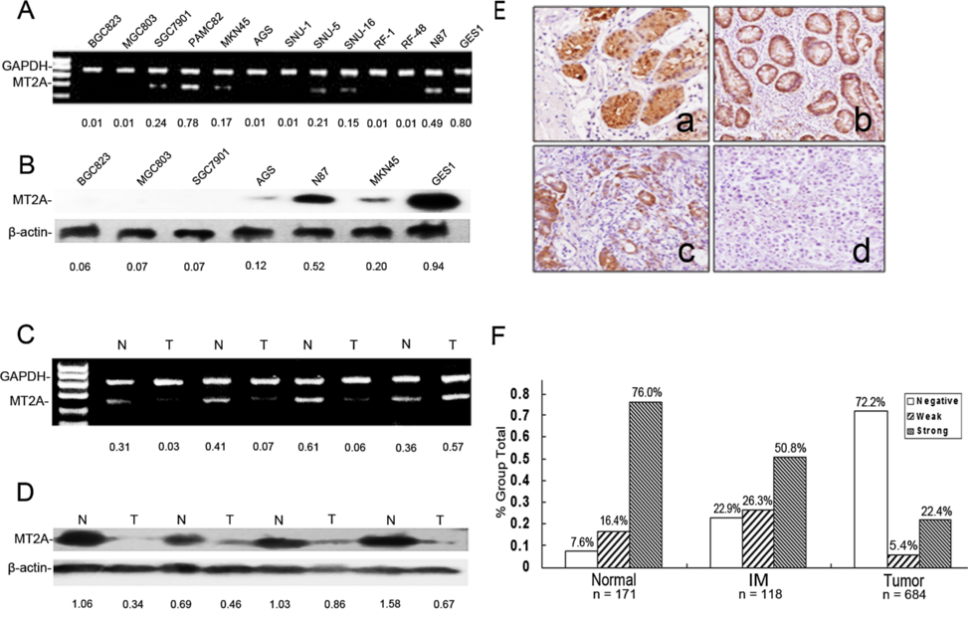
**Absent or decreased expression of MT2A is detected in cell lines and primary tumors of GC. A**, MT2A mRNA level was measured by RT-PCR in GC cell lines and the normal immortal cell line-GES1. And semi-quantification analyses were performed with Imagetool 3.0 gray scale scanning software (normalized to β-actin levels). The experiment was done in triplicate. **B**, MT2A protein level was determined by Western blot analysis in GC cell lines and the normal immortal cell line-GES1. The MT2A protein bands were scanned (normalized to β-actin levels). **C**, The differential expression of MT2A mRNA transcript in 36 paired GC tissues and adjacent normal tissues. The MT2A mRNA bands were scanned (normalized to β-actin levels). **D**, MT2A protein levels were detected in the 36 paired GC tissues by Western blotting. The MT2A protein bands were scanned (normalized to β-actin levels). **E**, Decreased MT2A was observed during gastric malignant transformation. **(a)** intense staining of MT2A in normal tissues, **(b)** moderate staining of MT2A in IM tissues, **(c)** weak staining of MT2A in moderately differentiated primary gastric cancers and **(d)** negative expression of MT2A in poorly differentiated GCs (original magnification × 400). **(D)** Compared with the normal and precancerous lesions-IM samples, reduced MT2A was a common molecular event in gastric carcinogenesis (*P* < 0.001) **F**: Differential expression of MT2A in GC, IM and normal tissues by IHC staining. Negative: score 0; Weak: score 1-4; Strong: score 5-12. For details, see the Additional file 1.

Expression of MT2A was evaluated by RT-PCR and Western blot in a panel of GC cell lines and GES1, which is served as “normal control”. As shown in Figure 1A, MT2A mRNA varied substantially with the highest levels in GES1. Lack or decrease of MT2A expression was observed in BGC823, MGC803, SGC7901, AGS, SNU-1, SNU-5, SNU-16, RF-1, RF-48 and N87 cells. The protein level of MT2A expression was further detected in the six common-used GC cell lines (BGC823, MGC803, SGC7901, AGS, N87, and MKN45) compared with GES1. Western blot analysis showed that, in the six commonly used GC cell lines, the levels of MT2A expression were absent or low compared with GES1, consistent with mRNA expression detected above (Figure 1B). Especially, BGC823, MGC803 and SGC7901 cells with lack of MT2A expression exhibit high tumorigenecity in nude mice.

36 primary tumors of GC with adjacent normal tissues were also examined in Figure 1C and D, to confirm the results derived from GC cell lines, decreased MT2A mRNA was displayed in GCs compared with that in matched normal controls (27/36, 75.0%, *P* < 0.001, Figure 1C). Low expression of MT2A protein was detected in 29 out of 36 GC cases (80.6%) compared with the matched normal tissues (*P* < 0.001, Figure 1D). The mRNA level of MT2A was correlated to the protein level detected by Western blot (R = 0.510, *P* = 0.009). In humans, the MTs are encoded by a family of genes consisting of 10 functional MT isoforms and the encoded proteins are conventionally subdivided into four groups, MT1, MT2, MT3, and MT4 proteins. Since the coding regions of MT isoforms are highly conserved in transcript and protein levels, MT2A is highly homologous with MT1G, MT1E and MT1F (Additional file 1: Figure S1B). Hence, experiments on any individual isoform should be carefully conducted to ensure that the exact isoform is analyzed. In this study, differential expression of MT1 transcripts was observed in GC cell lines and primary tumors of GC with adjacent normal controls (n = 36) (Additional file 1: Figures S1A and S2). We authenticated actual MT2A gene down-regulation in GC significantly compared with adjacent normal tissues (27/36, 75%, Figure 1C). There was no difference of MT1E and MT1F and MT1G mRNA transcripts in normal and tumor samples as well as MT1B mRNA transcript was not detectable in all cells and tissues (Additional file 1: Figures S1A and S2).

To investigate the candidacy of MT2A in gastric tumorigenesis, we initially characterized the status of MT2A expression in 171 normal tissues, 118 IM and 684 GC samples by IHC staining. The antibody for MT2A (E9 18-0133, Invitrogen) is not specific for MT-2A only, and cross-reacts with MT-1 to some extent, even if it is cloned by the full length of MT2A. Since there is no specific antibody to detect MT2A, so we use the common commercial antibody for IHC staining which was published in most related studies, MT2A expression is classified as low and high expression in normal appearance tissues, IM, and primary GCs. High level of MT2A expression was detected in 153 of 684 (22.4%) GC cases, and 60 of 118 (50.8%) IM cases, as well as 130 of 171 (76.0%) normal appearance cases, respectively (Additional file 1: Table S3, *P* < 0.001), it was consisted with MT2A mRNA transcripts and protein expression by Western blot. The expression of MT2A was classified as negative, weak and strong cases in 13 (7.6%), 28 (16.4%) and 130 (76.0%) out of 171 normal tissues, respectively; In IM tissues, 27 (22.9%), 31 (26.3%) and 60 (50.8%) out of 118 IM cases were detected respectively; In GC samples, 494 (72.2%), 37 (5.4%) and 153 (22.4%) out of 684 GC cases were detected respectively (Figure 1F, Additional file 1). There was a gradually decreased expression of MT2A protein in IM and GC (*P* < 0.001, Figure 1E and F). It is clear that MT2A mRNA was highly expressed in GES1 cells and normal tissues, but was reduced or lost in most GC cells and tissues. These results suggest that decreased MT2A is a molecular event in tumorigenesis and progression.

### MT2A expression is correlated with poor prognosis in GC

**Figure 2:**
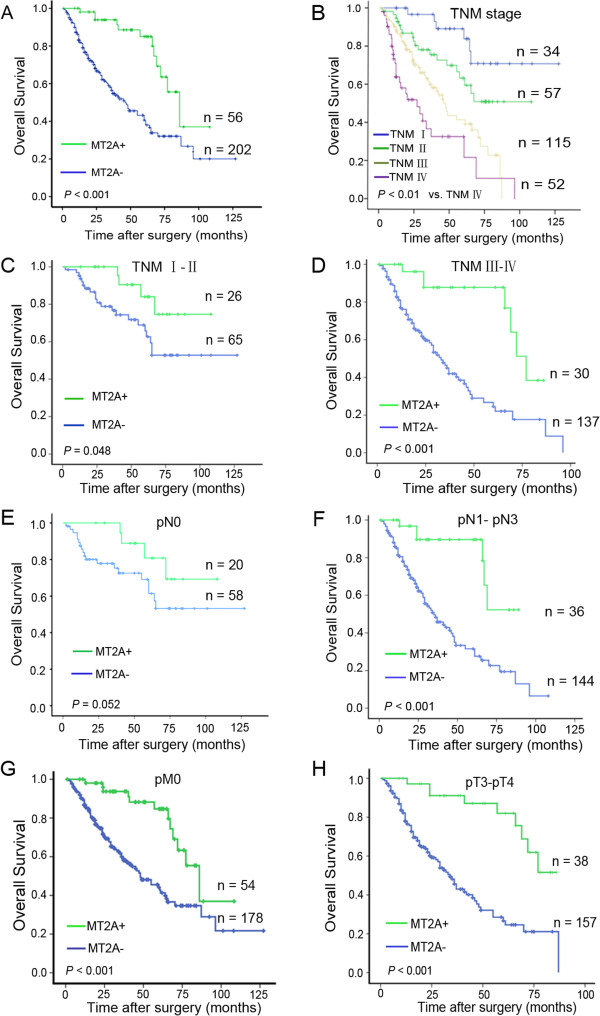
**Analysis of combined MT2A with TNM stage to predict clinical outcome in GC.** Kaplan–Meier analysis of overall-survival for MT2A expression was used according to TNM stage. **A**, Prognostic value of MT2A expression in GC patients was benefit for predicting the outcome of patients, low expression of MT2A was associated with poor overall survival (*P <* 0.01); **B**, TNM stage was benefit for the prognostic prediction in GC patients. **C D**, Prognostic value of MT2A in TNM stage I-II and stage III-IV (*P* = 0.048, *P <* 0.001, respectively); **E F**, Prognostic value of MT2A expression in lymph node metastasis or not (*P* < 0.001, *P* = 0.052, respectively); **G**. Prognostic value of MT2A expression in no distant metastasis subgroup (n = 232, *P* < 0.001); **H**. Prognostic value of MT2A in pT3-pT4 subgroup (*P* < 0.001). All data imply that MT2A could be an effective prognostic signature.

MT2A was frequently down-regulated in GC. To explore whether MT2A expression was a prognostic factor in human GC, MT2A protein expression was examined in the subset by IHC staining. The individual samples were categorized as low (MT2A-) or high expression (MT2A+). MT2A- was associated with poor overall survival (Figure 2A, *P* < 0.001). Univariate analysis indicated that some factors including decreased MT2A expression (*P* < 0.001), TNM stage (*P* < 0.01), tumor depth (*P* < 0.05), lymph node status (*P* < 0.05), distant metastasis (*P* < 0.001) and degree of differentiation (*P* = 0.011) were correlated with poor survival (Table 1). Cox multivariate analysis showed that the overall survival was associated with MT2A expression (MT2A+ versus MT2A- hazard ratio [HR], 0.429; 95% CIs, 0.187-0.683; *P* = 0.002, Table 2). We estimated the potential clinical significance of MT2A by correlating its expression pattern with the clinical parameters (Additional file 1: Table S4). MT2A exhibited an extensive positive correlation with tumor differentiation (*P* < 0.001) and inverse correlation with TNM stage (*P* = 0.05). These results indicate that MT2A is correlated with clinical pathological features and survival in GC patients.

**Table 2:** Cox regression analysis of clinicopathological features and molecular signatures in GC

	**Multivariate analysis**		
**Covariate by end point**	**Hazard ratio**	**95% CI**	***P-*value**
Age ≥ 60 years	1.300	0.840 to 1.934	0.254
Sex	0.823	0.499 to 1.233	0.292
Differentiation	0.753	0.509 to 1.778	0.875
Tumor invasive depth	1.914	1.389 to 2.638	< 0.001
Lymph node status	1.291	1.034 to 1.612	0.024
Distant metastasis	2.595	1.332 to 5.056	0.005
MT2A	0.429	0.187 to 0.683	0.002
IκB-α	0.779	0.524 to 1.311	0.423
p-IκB-α	1.857	1.256 to 2.912	0.003

### Prognostic significance of MT2A combined with TNM stage in GC patients

To further elucidate MT2A expression in prognostic significance, we combined MT2A expression with the clinicopathological features in GC. The aim was to evaluate the prognosis of GC patients with pathological classification, and to investigate whether pathological classification should be further sub-classified for more accurate prediction of outcome. As shown in Additional file 1: Table S4, MT2A expression in GC was associated with TNM stage and tumor differentiation, implying that MT2A may be a potential molecular biomarker to predict pathological classification in GC. As dichotomous covariates, both MT2A and TNM stage were independent predictors for survival (Table 2, Figure 2A and B). Prognostic significance of MT2A expression was further analyzed in GC patients according to the pTNM classification system. There was a significant difference in overall survival between patients with MT2A expression in both early (I and II) and advanced (III and IV) stage groups (*P* = 0.048 and *P* < 0.001, respectively; Figure 2C and D). Moreover, MT2A expression status was also effective for the prognosis in the same stages (*P* = 0.052, Figure 2E; *P* < 0.001, Figure 2F; *P* < 0.001, Figure 2G and *P* < 0.001, Figure 2H). These data represent that MT2A is a molecular signature to predict clinical outcome based on TNM stage.

### Restoration of MT2A expression results in growth suppression of GC cells *in vitro* and *in vivo*

**Figure 3:**
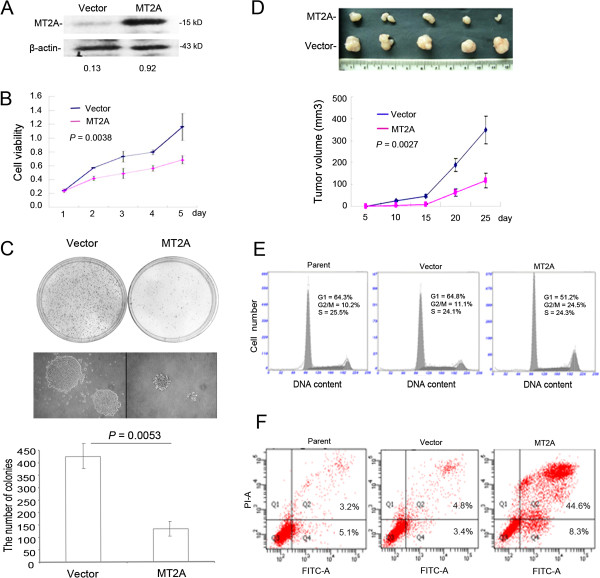
**Ectopic expression of MT2A inhibits GC cells growth *in vitro *and *in vivo.*** MT2A inhibited cell proliferation and colony formation in human GC cells. **A**, Western blot of MT2A was detected in BGC823 cells transfected with ectopic expression of MT2A plasmid and empty vector. The MT2A protein bands were scanned (normalized to β-actin to control for loading). **B**, Cell proliferation was analyzed by the MTT assay in re-expressing MT2A of BGC823 cells or not. Data was shown as the mean ± .SD of three independent experiments. **C**, Growth of BGC823 cells was analyzed in a semisolid soft agar medium. The empty vector colonies formed statistically significantly more colonies (mean = 426 large colonies per plate. 95% CIs: 387-472 colonies) than MT2A clones (mean = 136 colonies. 95% CIs: 100-164 colonies. The numbers represent the mean number of colonies of three independent experiments ± S.D. The relative number was calculated in control and BGC823-MT2A cells, and analyzed using Student’s *t*-test (*P* = 0.0038). **D**, MT2A-inhibited tumorigenicity of BGC823 cells in nude mice. The mean tumor volume for BGC823-MT2A at 3 weeks after injection was 112 mm^3^ (95% CIs: 93-147 mm^3^), compared with 352 mm^3^ (95% CIs: 298-423 mm^3^) for BGC823-Vector. Data represent the mean ± S.D of tumor volume derived from each group. In each group, tumor sizes were measured using a caliper at the indicated time points. Data were shown as mean ± S.D (*t-*test detection, *P* = 0.0027). **E**, FACS analysis showed that MT2A over-expression led to G2/M arrest, and the ratio of G2/M phase was higher than in empty control and parent cells (*P <* 0.01). **F**, Antitumor effect of MT2A was detected by annexin V/FITC-PI staining assay. Percentage of apoptotic cells was 52.9% ± 5.4% in MT2A group compared with that in the parent cells (8.3% ± 2.4% and cells transfected with the empty vector (8.2% ± 3.7%, *P* < 0.001).

To reveal the mechanism of MT2A in GC, we stably transfected MT2A over-expressed plasmid and empty vector into three GC cell lines (BGC823, SGC7901 and AGS). We examined the growth, apoptosis rates and cell cycle of these cell lines transfected with MT2A plasmid. Cell viability was reduced in GC cells re-expressing MT2A using MTT assay (BGC823, *P* = 0.0038, Figure 3B; AGS, *P* = 0.0007, Additional file 1: Figure S3A; SGC7901, *P* = 0.0004, Additional file 1: Figure S3C). To further investigate the function of MT2A in GC cell lines, we analyzed the effects of MT2A expression on cell proliferation and cell cycle regulation by Annexin-V/PI staining and flow cytometry analysis. More apoptosis was detected in these GC cells re-expressing MT2A (*P* < 0.01, respectively, Figure 3F; Additional file 1: Figure S3A and C). Furthermore, G2/M arrest was observed in GC cells using ectopic expression of MT2A. The ratios of G2/M phase in MT2A groups were twice higher than those in the vector groups did (*P* < 0.01, respectively, Figure 3E and Additional file 1: Figure S3B and D).

Further, we plated BGC823 cells repressing MT2A or not into soft agar, ectopic expression of MT2A was confirmed by western blot analysis first (Figure 3A). After 3 weeks of culture MT2A expression caused a statistically significant reduction in colony formation (*P* = 0.0053, Figure 3C). Furthermore, there was a dramatic growth inhibition of MT2A-re-expressing cells compared with the negative control (*P* = 0.0027, Figure 3D) in the xenograft models. These data demonstrate that MT2A might play a role in suppressing proliferation of GC cells *in vitro* and *in vivo*.

### MT2A represses NF-κB activity through IκB-α up-regulation

**Figure 4:**
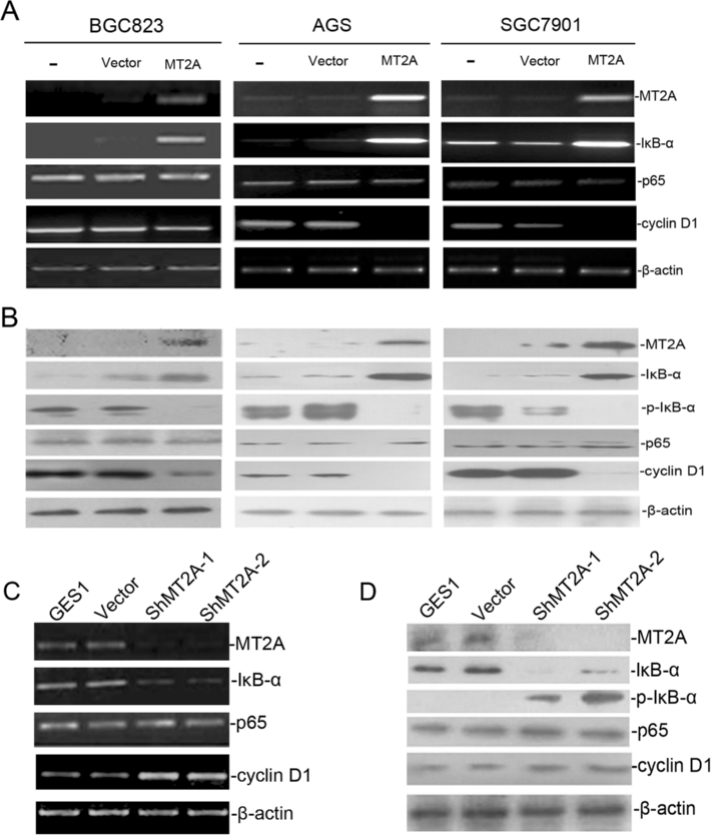
**MT2A was involved in regulating IκB-α expression in gastric cells.** MT2A positively regulated IκB-α expression. **A**, MT2A played a role in increasing IκB-α mRNA expression and reducing cyclin D1 mRNA expression in the NF-κB signaling but did not change p65 mRNA level in GC cells (“-”, parent cells). **B**, Western blot analysis showed that the level of MT2A expression was up-regulated and accompanied with elevation of IκB-α expression as well as decreased p-IκB-α and cyclin D1 in GC cells re-expressing MT2A. **C**, Silencing of MT2A in GES1 cells resulted in increased levels of cyclin D1 mRNA expression, as well as down-regulation of MT2A and IκB-α mRNA expression. **D**, cyclin D1 and p-IκB-α protein levels were increased by silencing of MT2A expression, accompanied with down-regulation of IκB-α expression.

**Figure 5:**
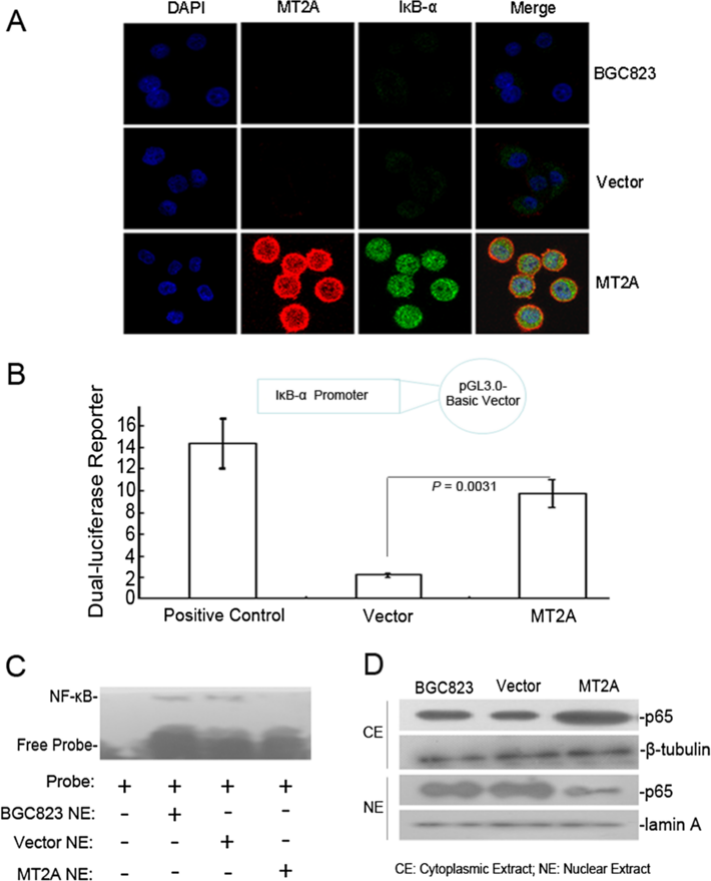
**MT2A mediated IκB-α transcript up-regulation to interfere NF-κB inactivation. A**, Increased IκB-α expression was induced by MT2A in BGC823 cells by immunofluorescence analysis. **B**, MT2A activated the promoter of IκB-α. 5.6-fold increase of IκB-α promoter luciferase activity was detected in MT2A group than that in Vector group. Data were presented relative to cells transfected with empty vector. Results represent mean ± S.D. for three independent experiments. Positive control means “PGL3-control (100 ng) + PRL-SV40 (10 ng)” plasmids are co-transfected in GC cells, and the high level of luciferase data will be detected and which evaluates the whole system of dual-luciferase activity. **C**, The nuclear translocation of NF-κB probe was decreased in BGC823 cells re-expressing MT2A by EMSA assay. **D**, The nuclear location of p65, as the main subunit of NF-κB, was reduced, and increased p65 exhibited in cytoplasm.

It was reported that the loss of MT2A induced NF-κB signal activation in transgenetic mice model (MT2A-knock out) [15]. But the role of MT2A as a friend or foe in NF-κB signal pathway was still controversial [18,33]. In order to interpret the mechanism of MT2A mediated GC cell growth suppression *in vivo* and *in vitro*, we tried to explore the potential relationship between MT2A and NF-κB signaling pathway, and the inner mechanism was still unclear in GC. MT2A and the main genes in NF-κB signaling, such as p65, IκB-α, p-IκB-α and the downstream effector of NF-κB signaling, cyclin D1 were detected in this study (Additional file 1). Ectopic expression of MT2A induced both mRNA and protein expression of IκB-α in all investigated GC cell lines (BGC823, SGC7901 and AGS), IκB-α mRNA steady-state levels were increased 5.5-fold in BGC823 cells re-expressing MT2A for 48 h. In AGS and SGC7901 cells, we observed a 7.2-fold and 4.3-fold induction of IκB-α mRNA (Figure 4A), the relative protein levels were consistent with mRNA transcripts, and the p-IκB-α protein was decreased in those GC cells transfected MT2A vector (Figure 4B). As shown in Figures 4C and D, knockdown of endogenous MT2A in GES1 cells led to down-regulation of IκB-α expression as well as up-regulation of p-IκB-α and cyclin D1 expression, suggesting a connection between MT2A activity and IκB-α expression. These experiments indicate that MT2A induces IκB-α expression in mRNA and protein levels. And MT2A-mediated up-regulation of IκB-α expression was further confirmed by immunofluorescence (Figure 5A, Additional file 1). To clarify the mechanism of MT2A on IκB-α expression, we performed luciferase reporter assays by restructuring of IκB-α promoter region, 5.6-fold up-regulation of IκB-α promoter luciferase activity was found in MT2A group than that in vector group (negative control) (*P* = 0.0035, Figure 5B). To investigate the relationship between MT2A and IκB-α-mediated NF-κB inactivation, we further studied the effects of MT2A in NF-κB nuclear translocation. As shown in Figure 5C, the DNA binding activity of NF-κB in nuclei was reduced by over-expressed MT2A. The protein levels and translocation of NF-κB main subunit p65 were detected in nuclear and cytoplasmic extracts derived from BGC823 cells transfected with MT2A or empty vector, and nuclear p65 was decreased in MT2A re-expressed group (Figure 5D, Additional file 1). Same results were observed in sections from BGC823 xenografts (Additional file 1: Figure S5A).

Based on the similarity of MT isoform, the specific primer pairs for different MT isoforms were designed to illustrate whether ectopic expression of MT2A or knockdown of MT2A could affect on other MT isoforms, as shown in Additional file 1: Figure S4, there was no obvious change for other MT isoforms after re-expression or knockdown of MT2A. Interestingly, different cell lines exhibited differential expression of MT isoforms. These data indicate that a significant reduction of p-IκB-α and cyclin D1 is induced by ectopic MT2A expression in GC cells, suggesting that MT2A suppressed cell proliferation and tumorigenicity through NF-κB inactivation.

### IκB-α expression is correlated with MT2A in gastric tumors

**Figure 6:**
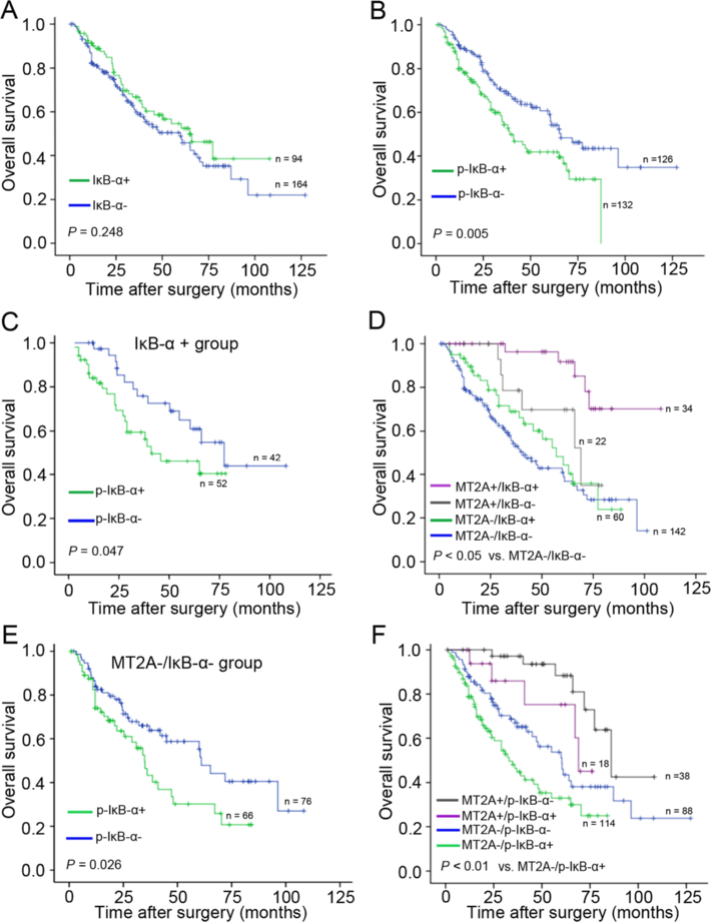
**Combined MT2A and IκB-α expression status as a prognostic indicator in GC. A**, IκB-α expression was not benefit for the clinical outcome (*P* = 0.248). **B**, The overall survival was better in subgroup with low expression of p-IκB-α (p- IκB-α-) than that in p-IκB-α + subgroup (*P* = 0.005). **C**, The expression of p-IκB-α status was benefit for prognostic prediction in the IκB-α + subgroup (n = 94, *P* = 0.047). **D**, Combination of MT2A and IκB-α was benefit for prognostic prediction in GC patients. MT2A+/IκB-α + subgroup had the better outcome than other subgroups (*P* < 0.05, respectively, other subgroups versus MT2A-/IκB-α-). **E**, In the MT2A-/IκB-α- subgroup, better survival was in the low level of p-IκB-α expression (n =142, *P* = 0.026). **F**, Combined MT2A and p-IκB-α expression together, is better for the prognostic prediction (*P* < 0.01, respectively, other subgroups versus MT2A-/p-IκB-α+).

To investigate the correlation between MT2A and IκB-α expression, we analyzed the sections of 684 GC, 118 IM and 171 normal tissues by IHC staining. As shown in Additional file 1: Table S3. Our data showed that both MT2A and IκB-α expression was absent in gastric malignancy. They had the positive correlation in the cohort (Spearman Coefficient = 0.429, *P* < 0.001, Additional file 1: Table S5). However, in the same cases, p-IκB-α (ser32/36) expression was up-regulated generally (Additional file 1: Figure S5B). Univariate survival analysis revealed that GC patients with high level of p-IκB-α expression exhibited poor survival (*P* = 0.005; Table 1 and Figure 6B). Multivariate analysis of p-IκB-α expression as covariates in multiple regression models showed that p-IκB-α expression was also the significant and independent prognostic factor when all variables were included in the multivariate regression equation. The relative risk for cancer-related death was increased in the subgroup with p-IκB-α + expression (95% CIs: 1.256-2.912, *P* = 0.003; Table 2), but the variation of IκB-α was not benefit for the clinical outcome (Figure 6A). These data indicate that decreased MT2A and IκB-α is involved in gastric malignant transformation.

### Combined MT2A and IκB-α expression status as a molecular signature to predict prognosis in GC

The aberrant NF-κB activation led to poor prognosis, associated with p-IκB-α activation and degradation of IκB-α in many types of cancer. To assess MT2A and IκB-α status in GCs in more detail, further stratification was conducted according to status of IκB-α expression. p-IκB-α + patients had shorter overall survival than p-IκB-α- patients in high expression of IκB-α (IκB-α+) subgroup (n = 94, *P* = 0.047, Figure 6C). When combining MT2A and IκB-α expression as the co-index for the prognostic prediction in GC, the overall survival was significantly better in MT2A+/IκB-α + group (*P* < 0.05, Figure 6D). In most samples with MT2A-/IκB-α- (n = 142), p-IκB-α + subgroup had the worse outcome (*P* = 0.026, Figure 6E). Furthermore, when combing MT2A and p-IκB-α expression together, we found that only MT2A+/p-IκB-α- group had the best survival (*P <* 0.01; Figure 6F). These results show that combination of MT2A and p-IκB-α expression might be a molecular signature to predict prognosis of GC.

## Discussion

GC is histopathologically heterogeneous and difficult for prognosis prediction by tumor grade or histological type. In this study, we offered both clinical and mechanistic evidence that MT2A is an independent prognostic factor and effective molecular target for cancer therapy. MT2A has been shown to reduce the tumorigenicity *in vivo* and *in vitro*, and decrease or loss of MT2A is a critical molecular event in GC cell lines and primary GC tissues. Decreased MT2A was associated with gastric malignant transformation, as well as poor survival. Re-expressing MT2A significantly inhibited the growth of GC cells. Interestingly, restoration of MT2A led to down-regulation of p-IκB-α and cyclin D1 but to induce IκB-α up-regulation, which was consistent with the typical apoptosis of GC cells resulting from suppression of NF-κB activation, accompanied with G2/M arrest [34-36]. Moreover, cyclin D1 is a therapeutic target in cancer. Its abundance will lead to oncogenic activation in stomach [37,38].

**Figure 7:**
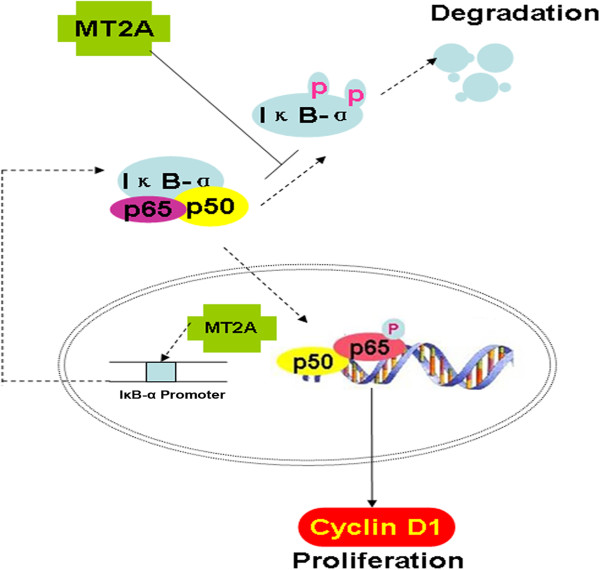
**The potential pathway of MT2A involved in NF-κB signaling.** MT2A mediated IκB-α up-regulation to suppress the NF-κB pathway activation. Ectopic expression of MT2A could induce upregulation of IκB-α in mRNA and protein levels, as well as down-regulation of p-IκB-α and cyclin D1expression. Moreover, MT2A could be one of the transcriptional factors to induce the activity of IκB-α promoter, which indicates that MT2A is the negative regulator in NF-κB signaling pathway.

Importantly, the luciferase activity of IκB-α promoter was induced by re-expressing MT2A in GC, leading to suppress the nuclear translocation of NF-κB. NF-κB has a key role as a pivotal link between inflammation and cancer [39]. Recently, NF-κB inhibitors have emerged as new therapeutic targets for neoplasia [23,40]. In this study, we have demonstrated MT2A expression is significantly related to apoptotic indices by suppressing NF-κB signaling activation (Figure 7). Mageed and Agrawal found a direct interaction of MT with the p50 subunit of NF-κB, which is a heterodimeric sequence-specific transcriptional activator [33]. Moreover, it has also been reported that MT is capable of modulating NF-κB transcriptional activity [18]. Some inverse results are reported that ApoMT (metal-free MT) has the potential to remove zinc from NF-κB and hence inactivate the NF-κB-mediated transcriptional activity consequent to zinc clelation [16]. MT possessed antioxidant properties that prevent H_2_O_2,_ or lipopolysaccharide (LPS)-stimulated NF-κB signaling in many inflammatory diseases [9,41-43].

Differential expression of MT isoforms was detected in GC cells and tissues, which indicated that the potential role of MT isoforms in carcinogenesis gained attention and make sure that the exact isoform is analyzed in our study. It is therefore not surprising that members of the MT family may be involved pleiotropically in a number of different biological functions except for ROS scavenger and metal-binding ability. However, there are no readily available commercial antibodies for distinguishing the highly homologous protein isoforms of MT1/2. The controversial results of MT in human neoplasia could possibly be attributed to the methods applied using antibodies that were unable to distinguish specific MT1/2 isoforms. In most studies where immunohistochemistry was applied, MT expression was revealed antibodies against a common epitope of MT1 and MT2A that were unable to detect over-expression due to other MT isoforms, reducing the significance of MT participation in tumors.

In addition, MT2A suppression is frequently observed in GC, and similar data was reported in hepatocellular and colon cancer [44,45]. Duncan et al reported that down-regulation of MT2A expression occurred upon immortalization, which implies that MT2A is down-regulated when human cells become immortal phenotypes, a key event in tumorigenesis [46].

Collectively, down-regulation of MT2A expression is an independent predictor for clinical outcome. It is conceivable that re-expression of MT2A can be considered as a molecular target in GC for molecular classification and individual therapy.

## Ethics approval

This study was conducted with the approval of Peking University Cancer Hospital & Institute Review Board.

## Competing interests

The authors declare that they have no competing interests.

## Authors’ contributions

YP: study concept and design; acquisition of data; analysis and interpretation of data; drafting of the manuscript; final approval of the version to be published. JH: study concept and design; analysis and interpretation of data; critical revision of the manuscript for important intellectual content; study supervision; final approval of the version to be published. RX, XY, JC, WL and JY: study concept and design; technical or material support; analysis and interpretation of data; critical revision of the manuscript for important intellectual content; study supervision; final approval of the version to be published. YL: study concept and design; analysis and interpretation of data; critical revision of the manuscript for important intellectual content; obtained funding; study supervision; final approval of the version to be published. All authors read and approved the final manuscript.

## Supplementary Material

Additional file 1: Table S1 Analysis of clinicopatholigical features and MT2A expression in GCs. **Table S2.** Oligonucleotide primers for RT-PCR. **Table S3.** Comparison of MT2A and IκB-α protein expression in tumors, intestinal metaplasia and normal tissues. **Table S4.** Relationship between MT2A and clinical features in GC. **Table S5.** Analysis of coexpressed MT2A and IκB-α in GC samples (n = 684). **Table S6.** Primers for MT isoforms. **Figure S1.** The similarity of MT1/2 isoforms and differential expression in GC cell lines. **Figure S2.** Differential expression of MT isoforms in paired GC tissues by RT-PCR and real-time PCR analysis. **Figure S3.** Ectopic expression of MT2A induced apoptosis and G2/M arrest in SGC7901 and AGS. **Figure S4.** The variation of other MT isoforms was detected in GC cells with re-expressed MT2A or knockdown of MT2A. **Figure S5.** MT2A and NF-κB signaling were detected in BGC823 xenografts with re-expression of MT2A and human gastric tissues by IHC staining. Click here for file
