# Supplementary material for: Metallothionein 2A inhibits NF-κB pathway activation and predicts clinical outcome segregated with TNM stage in gastric cancer patients following radical resection
Source: J Transl Med. 2013 Jul 19;11:173. doi: 10.1186/1479-5876-11-173 (PMC3726370; doi:10.1186/1479-5876-11-173)
Supplement: Additional file 1: Table S1 — Analysis of clinicopatholigical features and MT2A expression in GCs. Table S2. Oligonucleotide primers for RT-PCR. Table S3. Comparison of MT2A and IκB-α protein expression in tumors, intestinal metaplasia and normal tissues. Table S4. Relationship between MT2A and clinical features in GC. Table S5. Analysis of coexpressed MT2A and IκB-α in GC samples (n = 684). Table S6. Primers for MT isoforms. Figure S1. The similarity of MT1/2 isoforms and differential expression in GC cell lines. Figure S2. Differential expression of MT isoforms in paired GC tissues by RT-PCR and real-time PCR analysis. Figure S3. Ectopic expression of MT2A induced apoptosis and G2/M arrest in SGC7901 and AGS. Figure S4. The variation of other MT isoforms was detected in GC cells with re-expressed MT2A or knockdown of MT2A. Figure S5. MT2A and NF-κB signaling were detected in BGC823 xenografts with re-expression of MT2A and human gastric tissues by IHC staining. [file 1479-5876-11-173-S1.pdf]

Supplementary Materials and Methods

RNA isolation and RT-PCR from fresh tissues and cell lines

Total RNA was isolated from the cultured cells and powdered tissue according to the protocol supplied with TRIzol Reagent (Invitrogen, Life Technologies, Carlsbad, CA) according to the manufacturer's instructions. The concentration and purity of the RNA samples were determined using spectrophotometer scan in the ultraviolet (UV) region and ethidium bromide (EB) visualization of intact 18S and 28S RNA bands after agarose gel electrophoresis. Total RNA (2 μg) was reverse transcribed (RT) with MMLV reverse transcriptase (50 U, Invitrogen Life Technologies, Carlsbad, CA) in 10×polymerase chain reaction (PCR) buffer (500 mM KCl and 100 mM Tris-HCl, pH 8.3), 5 mM $MgCl_2$, 20 U RNase inhibitor, 1 mM each of the dNTPs, and 2.5 μM random hexanucleotide primers). Primers were listed in the Supplementary Table 2 and 6. The final PCR products were electrophoresed on 1.5% agarose gels containing EB along with DNA markers. The intensity of the PCR product bands was determined on a Dell workstation configured with Kontron KS 400 image-analysis software.

Real-time PCR

The expression of MT isoforms and β-actin mRNAs was determined using real-time PCR. Each cDNA sample was amplified using SYBR Green (TransGen Biotech Co., Ltd. Beijing) on the ABI 7500 Fast Real-time PCR System (Applied Biosystem, CA). Briefly, the reaction conditions consisted of 2 μl of cDNA and 0.2 μM primers in a final volume of 20 μl of supermix. Each cycle consisted of denaturation at 95°C for 15s, annealing at 58.5°C for 5s and extension at 72°C for 10s, respectively. The primers for MT1/2 isoforms were listed in Supplementary Table 6. β-actin was used as an endogenous control to normalize each sample. The experiment was performed by three independent experiments with triplicate.

Transfection

Full-length MT2A cDNA was cloned into pcDNA3.1 (-) vector to construct pcDNA3.1 (-)-MT2A recombinant plasmid (Supplementary Table 2). Stable transfection and over-expression of MT2A in BGC823 were measured by Western Blot and RT-PCR, respectively. Knockdown of MT2A was studied in GES1 cells by shRNA, and the sequences of shMT2A-1 and shMT2A-2 were listed in Supplementary Table 2. The promoter of IκB-α gene luciferase reporter construct was cloned into BglII/HindIII sites in the pGL3-Basic reporter plasmid (Promega, Madison, WI, USA) (Supplementary Table 2).

Immunoblot analysis

Immunoblot analysis was performed using antibodies against MT2A (1:600 dilution; ab12228, Abcam, UK), NF-κB p65 (1:600 dilution; sc-8008, Santa Cruz, CA), IκB-α (1:800 dilution; sc-371, Santa Cruz), p-IκB-α (ser32/36) (1:1000 dilution; ab47752, AbCam, Cambridge, UK), cyclin D1 (1:500 dilution, sc-753, Santa Cruz) and β-actin (1:10000 dilution, A2228, Sigma, St Louis, MO). Species-specific HRP-labeled secondary antibodies were then added. The blots were visualized using enhanced chemiluminescence (Amersham Biosciences) and quantitated.

Immunohistochemical analysis

In clinical samples, MT2A, IκB-α and p-IκB-α were scored following a semi-quantitative scale (- to +++) by evaluating in representative tumor areas the intensity and percentage of cells showing significantly higher immunostaining than control cells in normal gastric tissues. Nuclear and cytoplasmic immunostaining in tissues was considered positive staining for MT2A and IκB-α, while, nuclear staining in tissues was considered positive staining for p-IκB-α. MT2A and IκB-α were scored based upon

percentage of cells showing cytoplasmic and nuclear staining (0 = 0%; 1 = 1-25%; 2 = 26-50%; 3 = 51-75%; 4 = 76-100%) and intensity of the staining (1 = mild; 2 = moderate; 3 = intense); p-IκB-α was scored for nuclear staining and intensity of staining. The scores of each tumor sample were multiplied to give a final score of 0-12, and the tumors were finally determined as negative (-), score 0; lower expression (+), score 1-4; moderate expression (++), score 5-8; and strong expression (+++), score ≥ 9. Tumor samples scored (++) to (+++) were considered as “high expression”, and cases scored (-) and (+) were termed as “negative” and “weak expression”, respectively. Meanwhile, samples scored (-) to (+) were considered as “low expression”.

Fluorescence microscopy assay

For immunofluorescence assay, stably transfected GC cells were seeded onto cover-slips in a cell culture dish. Experiments were terminated after 24 h by fixing cells with 4% paraformaldehyde for 10 min. After washed with PBS, cells were incubated with anti-MT2A (1:50 dilution; ab12228), and anti-IκB-α (1:100 dilution; sc-371, Santa Cruz) at 4°C overnight, then incubated with Fluorescein isothiocyanate or Rhodamine conjugated-secondary antibody (1:50 dilution; Santa Cruz) for 1 h, and stained nuclei with DAPI (Invitrogen, Life Technologies, Grand Island) for 2 min. Images of cells with fluorescent signal were captured by laser confocal microscopy (Leica Sp5 Laser Scanning Confocal Microscope, GE).

Electrophoretic mobility shift assay (EMSA)

Nuclear extracts of whole cells were prepared by the method of Applygen Inc (Applygen Technologies Inc, Beijing, China). Nuclear extracts of GC cells transfected with MT2A over-expressed plasmid or empty vector were prepared as followed the instruction described. Purity of nuclear extracts was confirmed by

assessing the relative level of lamin A in nuclear protein. Protein concentrations were determined by bicinchoninic acid assay with trichloroacetic acid precipitation using BSA as a reference standard (Pierce, Rockford, IL). Double-stranded NF-κB consensus oligonucleotide (5'-AGTGAGGGGACTTTCCC AGGC-3', Sangong, Ltd., Shanghai) was 5'-end labeled with biotin. Binding reactions containing equal amounts of protein (50 ng of oligo-nucleotides were performed for 30 min in binding buffer (4% glycerol, 1 mM $MgCl_2$, 0.5 mM EDTA, pH8.0, 0.5 mM dithiothreitol, 50 mM NaCl, 10 mM Tris, pH 7.6, 50 μg/ml poly [dI-dC]; Pharmacia LKB Biotechnology Inc., Piscataway, NJ). Reaction volumes were held constant to 15 μl. Reaction products were separated in a 7% poly-acrylamide gel and analyzed by autoradiography.

Luciferase activity assay

PGL-3.0 basic construct (Promega, Madison, WI) containing IκB-α promoter region sequence, with an internal control (0.01 μg/well pRL-TK Renilla luciferase vector, Promega), were cotransfected with pcDNA3.1-MT2A or empty vector (0.1μg/well) into BGC823 cells in a 96-well plate, using Lipofect 2000 (Invitrogen, Life Technologies). Transfection was carried out in triplicate. Forty-eight hours posttransfection, the cells were washed with PBS, and lysed in 1 × passive lysis buffer (Dual Luciferase Kit; Promega). The cell lysates were transferred into an OptiPlate 96-well plate (Perkin-Elmer, Waltham, Massachusetts) and assayed in a 1420-Multilabel counter luminometer, VICTOR (Perkin-Elmer) using the Dual-Luciferase kit (Promega). Relative luciferase units were measured and normalized against Renilla luciferase activity. Data were expressed as a mean of triplicate values (± SD) of the normalized PGLFLASH activity of pcDNA3.1-MT2A relative to the vector transfected cells (set as 100%)

Supplementary Tables

Supplementary Table S1

Analysis of clinicopathological features and MT2A expression in GCs

Characteristics	Cohort		Subset	
	No.	%	No.	%
Total	684		258	
Sex				
Male	513	75.0	186	72.1
Female	171	25.0	72	27.9
Age at diagnosis				
Median	54		50	
<60	391	57.2	173	67.0
≥60	293	42.8	85	33.0
TNM stage				
I	76	11.1	34	13.2
II	123	18.0	57	22.1
III	330	48.2	115	44.6
IV	155	22.7	52	20.1
Tumor depth				
pT1	32	4.7	13	5.0
pT2	107	15.6	50	19.4

pT3	435	63.6	157	60.9
pT4	110	16.1	38	14.7
Lymph node status				
pN0	176	25.7	78	30.2
pN1	344	50.3	115	44.6
pN2	133	19.4	49	19.0
pN3	31	4.5	16	6.2
Distant metastasis				
pM0	602	88.0	232	90.0
pM1	82	12.0	26	10.0
Degree of differentiation				
D1	142	20.8	54	20.9
D0	542	79.2	204	79.1
MT2A expression				
High	153	22.4	56	21.7
Low	531	77.6	202	78.3

M0: no distant metastasis; M1: distant metastasis;

D1: well /moderately-differentiated GCs; D0: poorly-differentiated GCs.

Supplementary Table S2. Oligonucleotide primers for RT-PCR

Target gene (length)	Accession Number	Primer ID	Sequence (5'-3')
MT2A (242 bp)	NM_005953	Forward	TCGCCATGGATCCCAACTG
		Reverse	AGGTTTGTGGAAGTCGCGT
GAPDH (472 bp)	NM_002046	Forward	ACCACAGTCCATGCCATCAC
		Reverse	TCCACCACCCTGTTGCTGTA
β-actin (513 bp)	NM_001101.3	Forward	CGGGAAATCGTGCGTGACATT
		Reverse	CTAGAAGCATTTGCGGTGGAC
p65 (333 bp)	NM_021975.3	Forward	GGCGAGAGGAGCACAGATACCA
		Reverse	CCGCACAGCATTCAGGTCGTA
IκB-α (197 bp)	NM_020529.2	Forward	CGTCTT ATTGTGGTAGGATCAGC
		Reverse	ACCACTGGGGTCAGTCACTC
Cyclin D1 (189 bp)	NM_053056	Forward	GAGGCGGAGGAGAACAAACA
		Reverse	GAAGCGTGTGAGGCGGTAG
**Primers used for construct**			
MT2A	NM_005953	Forward	CTGCTCGAGCACGCCTCCTCCAAG TC
		Reverse	CTGAAGCTTTAGCAAACGGTCACGGTC
**ShRNA for MT2A Psilencer3.1-H1-Neo**			
MT2A	NM_005953	ShMT2A-F1	GATCCGCCGTGACCGTTTGCTATATTT CAAGAGAATATAGCAAACGGTCACGG TTTTTTGGAAA

		ShMT2A-R1	AGCTTTTCCAAAAAACCGTGACCGTT TGCTATATTCTCTTGAAATATAGCAAA CGGTCACGGCG
MT2A	NM_005953	ShMT2A-F2	GATCCAGAACGCGACTTCCACAAATT CAAGAGATTTGTGGAAGTCGCGTTCT TTTTTTGGAAA
		ShMT2A-R2	AGCTTTTCCAAAAAAAGAACGCGAC TTCCACAAATCTCTTGAATTTGTGGA AGTCGCGTTCTG
IκB-α promoter luciferase reporter	NC_000014.8	Forward	GGA AGA TCTGAA AGC AAATCCCTA
		Reverse	CCCAAGCTT CGG ACG AGCTGCGGG

Supplementary Table S3

Comparison of MT2A and IκB-α protein expression in tumors, intestinal metaplasia and normal tissues

		MT2A		IκB-α	
Histology	Total cases	High (%)	Low (%)	High (%)	Low (%)
Tumor	684	153 (22.4)	531 (77.6)	189 (27.6)	495 (72.4)
IM	118	60 (50.8)	58 (49.2)	56 (47.5)	62 (52.5)
Normal	171	130 (76.0)	41 (24.0)	135 (79.0)	36 (21.0)

MT2A protein or IκB-α protein expression was obviously different in Normal vs. IM, $P < 0.001$; Tumor vs. Normal, $P < 0.001$; Tumor vs. IM, $P < 0.001$.

Supplementary Table S4

Relationship between MT2A and clinical features in GC

		MT2A		
Characteristics	No. of cases	High (%)	Low (%)	*P*-value
Total	258			
Sex				
Male	186	39 (21.0)	147 (79.0)	
Female	72	17 (23.6)	55 (76.4)	0.644
Age at diagnosis				
<60	173	37 (21.4)	136 (78.6)	
≥60	85	19 (22.4)	66 (77.6)	0.857
TNM stage				
I	34	8 (23.5)	26 (76.5)	
II	57	18 (31.6)	39 (68.4)	
III	115	25 (21.7)	90 (78.3)	
IV	52	5 (9.6)	47 (90.4)	0.05
Tumor depth				
pT1-pT2	63	18 (28.6)	45 (71.4)	
pT3-pT4	195	38 (19.5)	157 (80.5)	0.128
Lymph node status				
pN0	78	20 (25.6)	58 (74.4)	
pN1-pN3	180	36 (20.0)	144 (80.0)	0.313

Distant metastasis				
pM0	232	54 (23.3)	178 (76.7)	
pM1	26	2 (7.7)	24 (92.3)	0.08
Degree of differentiation				
D1	54	22 (40.7)	32 (59.3)	
D0	204	34 (16.7)	170 (83.3)	< 0.001

pM0: no distant metastasis; pM1: distant metastasis;

D1: well /moderately-differentiated GCs; D0: poorly-differentiated GCs;

Supplementary Table S5

Analysis of coexpressed MT2A and IκB-α in GC samples (n = 684)

	IκB-α		
MT2 A	Positive Case (%)	Negative Case (%)	*P*-value
Positive Case	97 (63.4)	56 (36.6)	< 0.001
Negative Case	92 (17.3)	439 (82.7)	

Spearman Correlation Coefficient = 0.429

Supplementary Table S6 Primers

Gene (Accession Number)	Primer (5'-3')	Size	From-To	TM
MT2A	F:TCGCCATGGATCCCAACTG	242	86-104	60.6
(NM_005953)	R:AGGTTTGTGGAAGTCGCGT		327-309	
MT1A	F: GTGCGCCTTATAGCCTCTCA	225	15-34	60.2
(NM_005946.2)	R:TCTCTGATGCCCCTTTGCAG		239-220	
MT1B	F:GGCTCCAAATGGATCCCAAC	180	48-67	59.0
(NM_005947.2)	R:GGCACTTCTCTGATGAGCCT		227-208	
MT1E	F:CTGCCTGACTGCTTGTTCGT	259	123-142	60.5
(NM_175617.3)	R:AAGAGCTGTTCCCACATCAGG		381-361	
MT1F	F:CCCTCCCCTGACTATCAAAGC	283	4-24.	59.8
(NM_005949.3)	R:CACTTCTCTGACGCCCCTTT		286-267	
MT1G	F:TTCCCTTCTCGCTTGGGAAC	258	31-50	60.1
(NM_005950.1)	R:TTTGTACTTGGGAGCAGGGC		288-269	
MT1H	F:CTTCTCGCTTGGGAACTCCA	257	34-53	59.5
(NM_005951.2)	R:CTGTTTTCATCTGACAGCAGGG		290-269	
MT1X	F:CTGCTTCTCCTTGCCTCGAA	186	52-71	60.1
(NM_005952)	R:TGTCTGACGTCCCTTTGCAG		237-218	
MT3	F:CAAGCGCACAAACGGAAAGAG	187	60-80	
(NM_005954.2)	R:CAGGGTCCATGTCGAGGAGA		246-227	60.8
MT4	F: GGAGGAATCTGCATGTGTGGA	141	111-131	
(NM_032935.2)	R: CTTGTCTGAGCCTCCTTTGC		251-232	59.0
β-actin	F: TTAGTTGCGTTACACCCTTTC	150	1225-1245	
(NM_001101.3)	R: ACCTTCACCGTTCCAGTTT		1374-1356	59.5

Supplementary Figure Legend

Supplementary Fig.1

The similarity of MT1/2 isoforms and differential expression in GC cell lines

A, The differential expression of MT1/2 isoforms in GC cells as well as GES1; B, Multiple alignments and phylogenetic tree were constructed from nucleotide sequences of human MT family. Sequences were aligned with the DNAman 3.0 program. Especially, MT2A, MT1E, MT1F, MT1G and MT1H are similar in mRNA transcripts (left); Multiple alignments and phylogenetic tree were constructed from amino acid sequences of human MT family. Sequences were aligned with the Clustal X 1.8 program. Some conserved amino acid residues represent in all putative MT proteins. Especially, MT2A, MT1G, MT1E, MT1F and MT1A are similar in protein level.

Supplementary Fig.2

Differential expression of MT isoforms in paired GC tissues by RT-PCR and real-time PCR analysis

A, Differential expression of MT isoforms was exhibited in primary gastric tumors and adjacent normal tissues by RT-PCR. B, No obvious change for each MT1/2 isoform was observed in matched GCs compared with normal controls by real-time PCR.

Supplementary Fig.3

Ectopic expression of MT2A induced apoptosis and G2/M arrest in SGC7901 and AGS

A, MT2A induced AGS cells apoptosis by Annexin V/PI staining. And the cell proliferation was used by MTT assay for 5 days. More apoptosis was detected in these GC cells re-expressing MT2A. A, AGS-MT2A vs. AGS-Vector: 66.5% ± 7.2% vs. 20.9% ± 5.6%, P = 0.0074; SGC7901-MT2A vs.

SGC7901-Vector: 53.8% ± 8.9% vs. 10.0% ± 6.4%, $P = 0.0026$. B, D, G2/M arrest was induced in AGS and SGC7901 re-expressing MT2A ($P < 0.01$, respectively).

Supplementary Fig.4

The variation of other MT isoforms was detected in GC cells with re-expressed MT2A or knockdown of MT2A

Expression changes of other MT isoforms were failed to detect in those two cell lines SGC7901 and AGS re-expressed MT2A or knockdown of MT2A.

Supplementary Fig.5

MT2A and NF-κB signaling were detected in BGC823 xenografts with re-expression of MT2A and human gastric tissues by IHC staining

MT2A and the important genes of NF-κB signaling were detected in BGC823 xenografts by IHC staining (original magnification ×200). B, the same trend of MT2A and IκB-α protein expression was decreased during gastric malignancy, but p-IκB-α was inversely activated in the same samples (original magnification ×400).

Supplementary Fig. 1

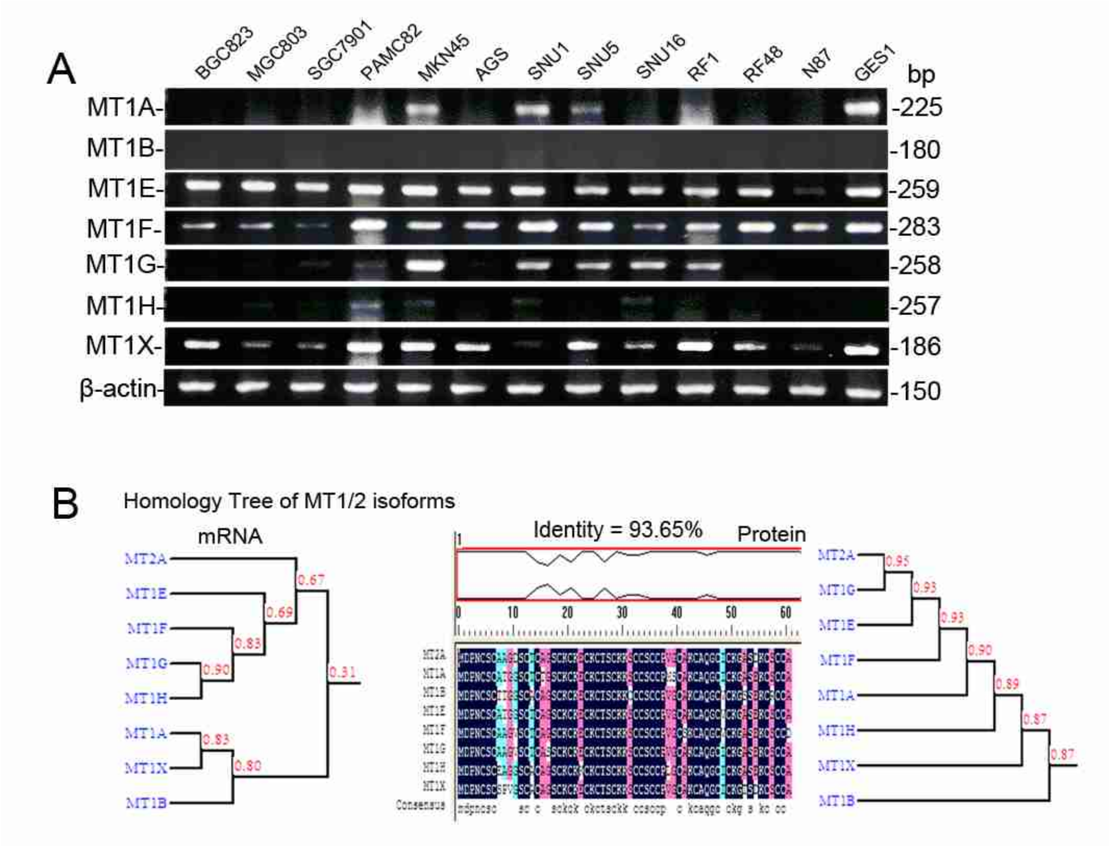

Supplementary Fig. 2

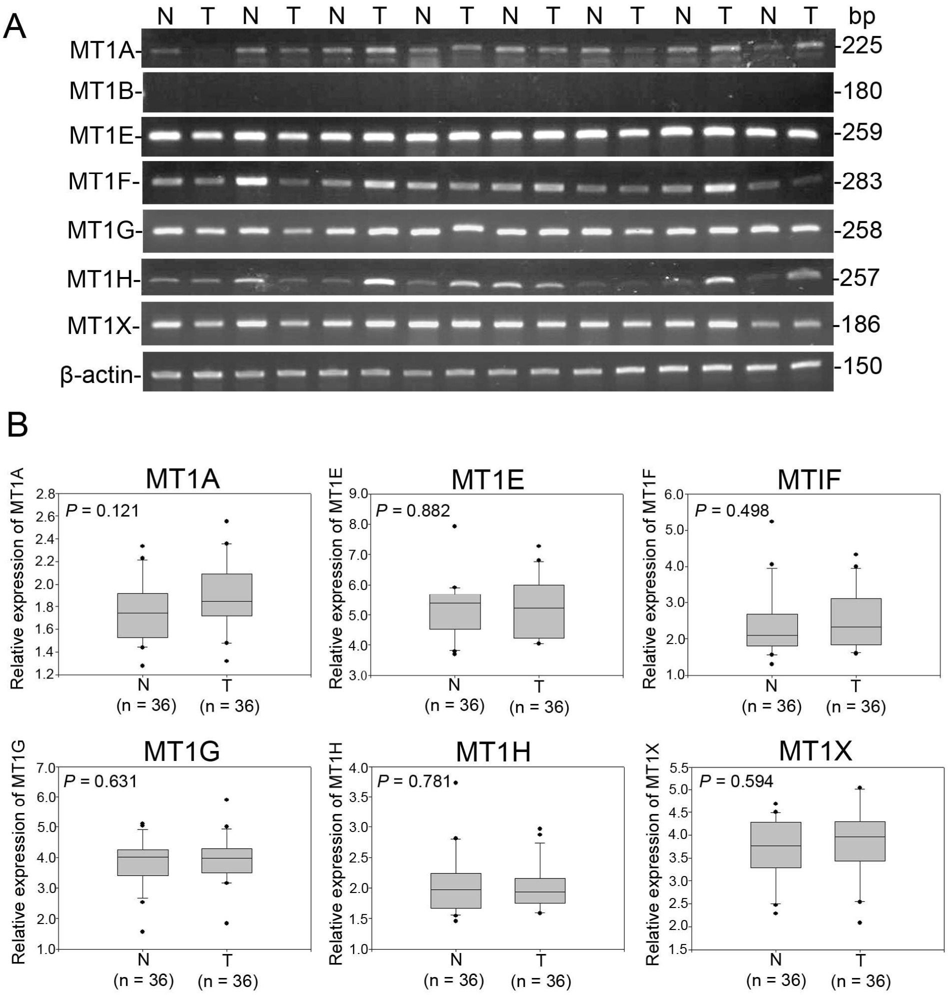

Supplementary Fig.3

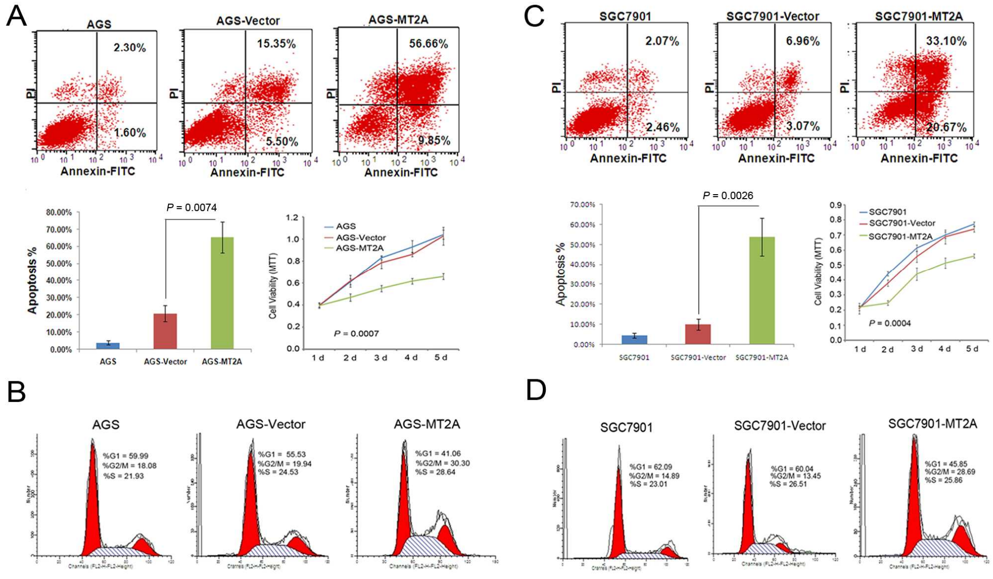

Supplementary Fig. 4

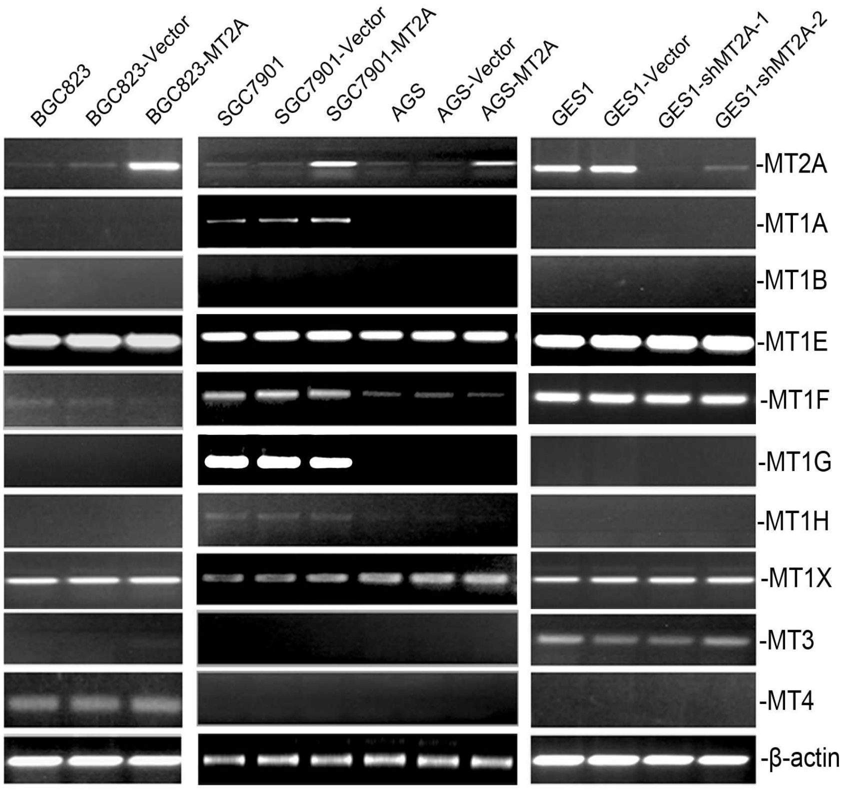

Supplementary Fig. 5

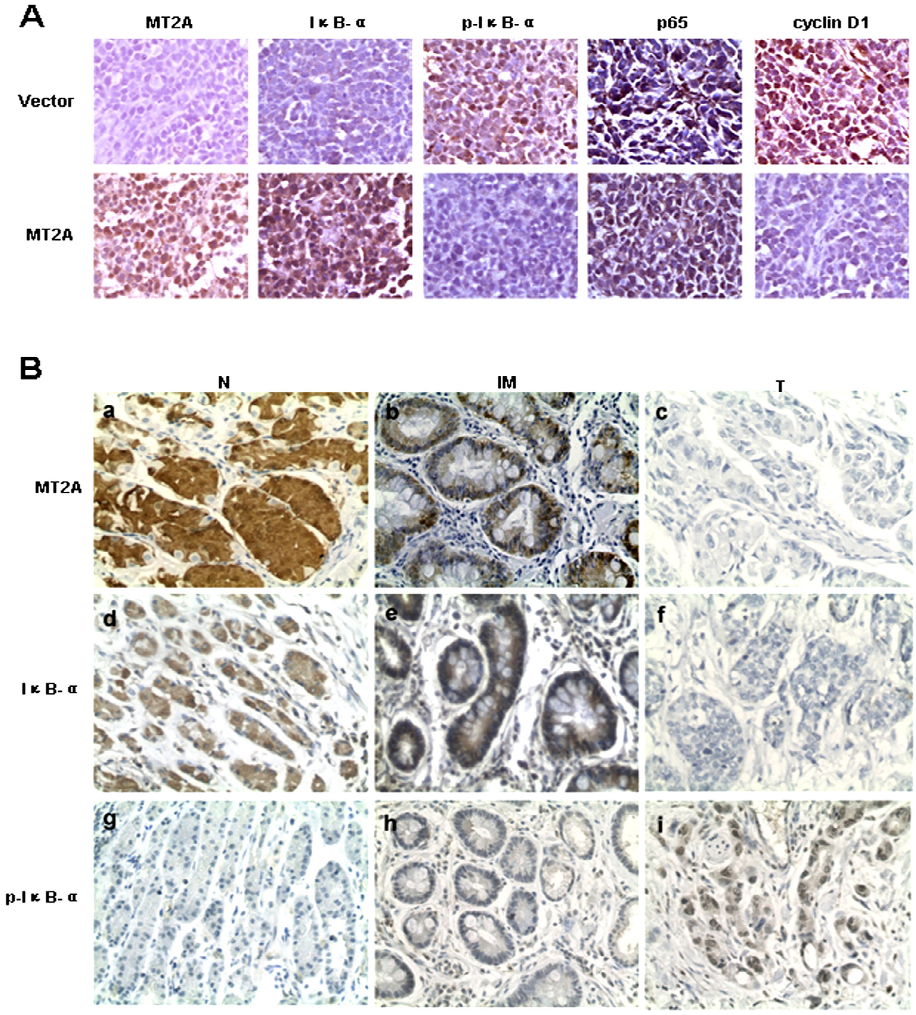

Certification for Medical or Scientific Journal Editor from Ethics Committee of The School of Oncology, Peking University (Beijing Cancer Hospital)

To editors of Journal of Translational Medicine,

Article, "Metallothionein 2A interferes NF-κB pathway activation and predicts clinical outcome segregated with TNM stage in gastric cancer patients following radical resection" written by Yuanming Pan, Jiaqiang Huang and Youyong Lu. The investigation project and its informed consent from have been examined and certified by Ethics Committee of Beijing Cancer Hospital.

Ethics Committee of
The School of Oncology, Peking University
(Beijing Cancer Hospital)
28th February, 2011

地址：北京市海淀区阜城路 52 号
邮编：100142
电话：0086-10-88196391

No.52 Fu-cheng Road, Haidian District, Beijing
Beijing 100142, P.R. China
Tel: 0086-10-88196391
